# Supplementary figures and images for: Time-restricted feeding has a limited effect on hepatic lipid accumulation, inflammation and fibrosis in a choline-deficient high-fat diet-induced murine NASH model
Source: PLoS One. 2024 Jan 29;19(1):e0296950. doi: 10.1371/journal.pone.0296950 (PMC10824409; doi:10.1371/journal.pone.0296950)

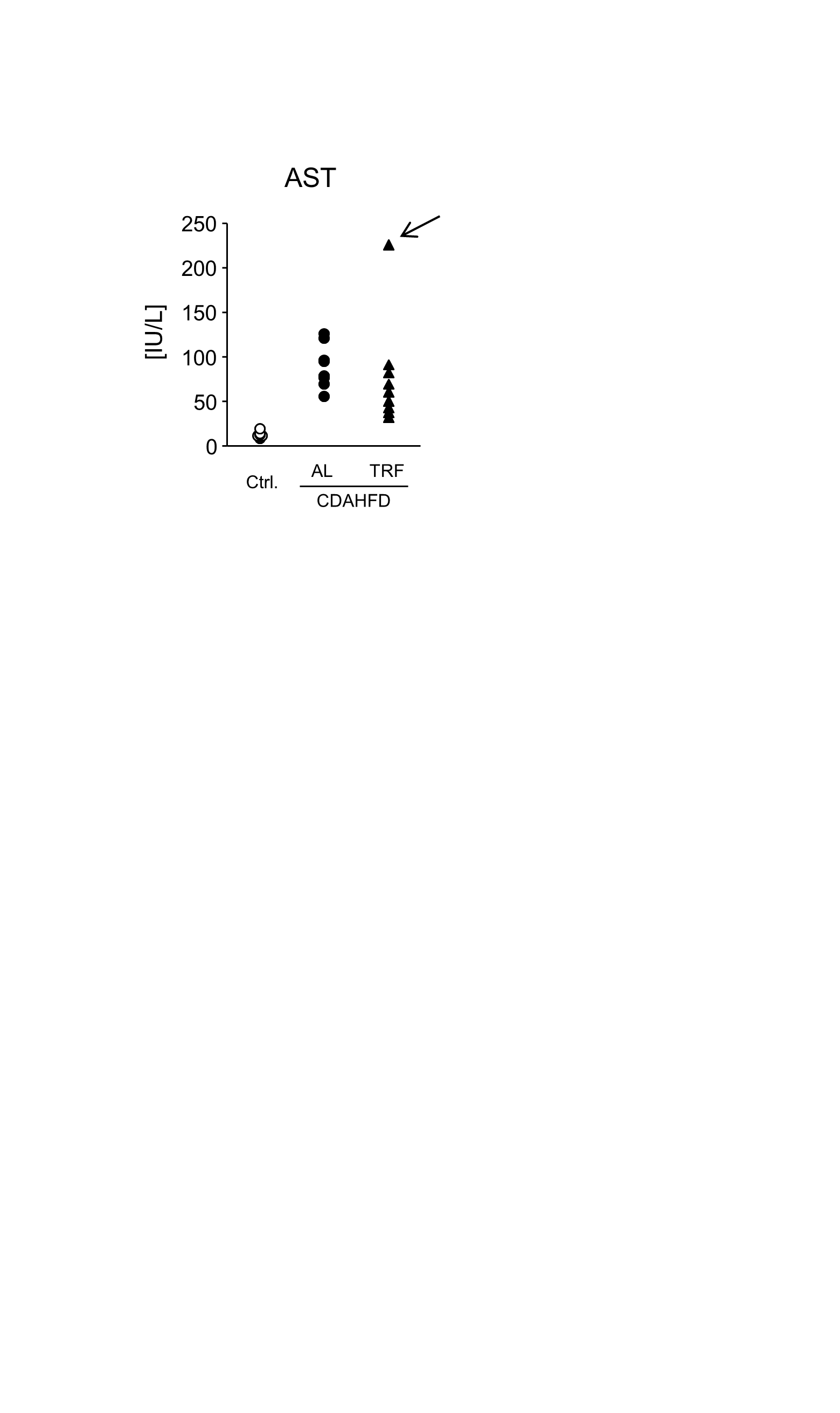

Supplement: S1 Fig — Plasma AST levels were widely distributed in the TRF group. This is mainly owing to the relatively high value indicated by an arrow. The mouse depicted in this figure exhibited mild cirrhosis and a high AST/ALT ratio (2.80), indicating prominent progression of fibrosis [29]. When this sample was eliminated, a statistically significant difference between AL and TRF was detected (P<0.01) using a one-way ANOVA followed by Tukey’s multiple comparison test. (TIF) [file pone.0296950.s001.tif]

$\alpha$ SMA

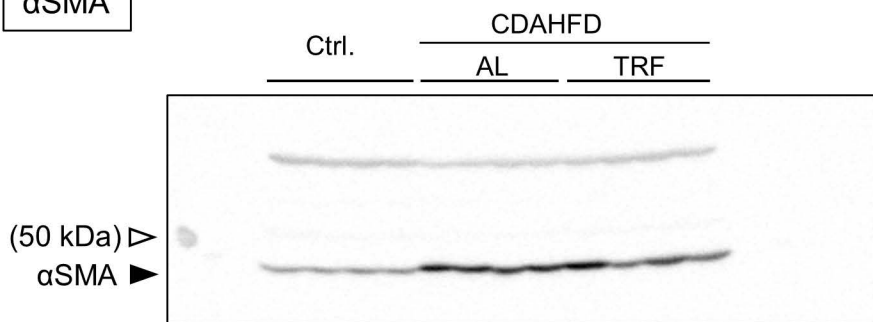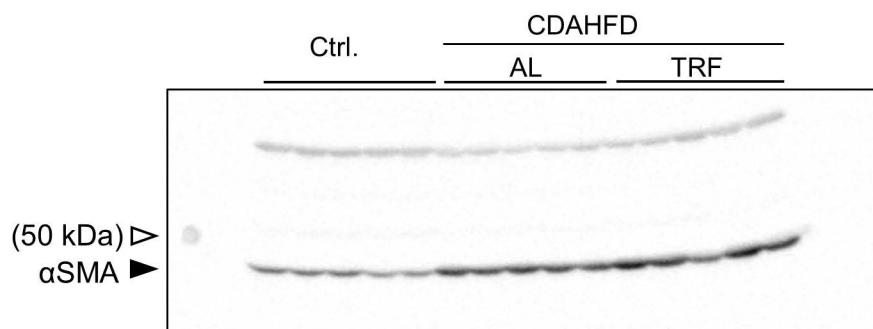

CBB staining

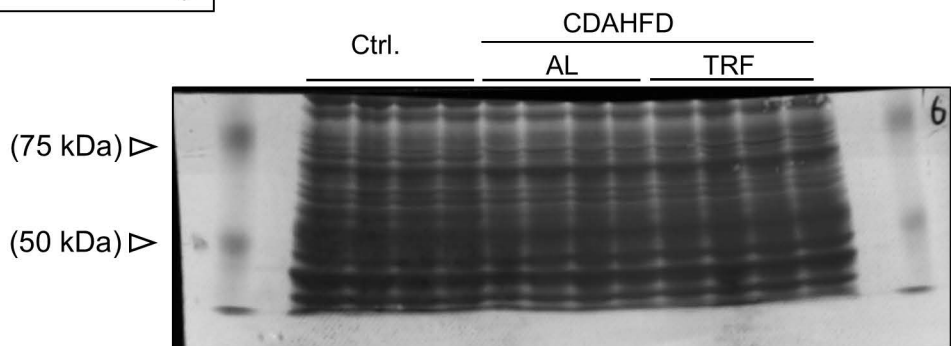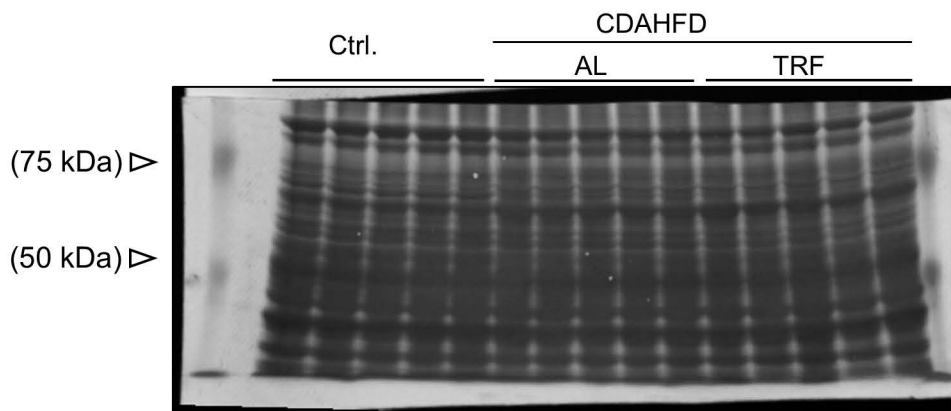

Supplement: S1 Raw images — Mice were fed CE-2 diet (Ctrl) or CDAHFD ad libitum (AL) or CDAHFD during active phase between ZT14-22 (TRF) for 8 weeks. Raw images of western blot for αSMA and CBB staining of liver proteins are shown. (PDF) [file pone.0296950.s002.pdf]
