# Supplementary material for: Time-restricted feeding has a limited effect on hepatic lipid accumulation, inflammation and fibrosis in a choline-deficient high-fat diet-induced murine NASH model
Source: PLoS One. 2024 Jan 29;19(1):e0296950. doi: 10.1371/journal.pone.0296950 (PMC10824409; doi:10.1371/journal.pone.0296950)
Supplement: Attachment — Submitted filename: Response_to_Reviewers.docx [file pone.0296950.s003.docx]

PONE-D-23-31949

Time-restricted feeding has a limited effect on hepatic lipid accumulation, inflammation and fibrosis in choline-deficient high-fat diet-induced murine NASH model

PLOS ONE

Dear Dr. Sato,

Thank you for submitting your manuscript to PLOS ONE. After careful consideration, we feel that it has merit but does not fully meet PLOS ONE’s publication criteria as it currently stands. Therefore, we invite you to submit a revised version of the manuscript that addresses the points raised during the review process.
**More specifically, authors should critically revise statements regarding the proposed model as a proxy to human lean NAFLD, as also requested by one reviewer. Moreover, authors report food consumption per mouse: in fact, they never measured this parameter in individual mice, as animals were (presumably) housed in groups (although this is never stated in the　MS).　Reference to this issue should be made clear in the text.**

**Finally, language should be carefully checked throughout the MS.**

**Response**

**We deleted statements regarding the CDAHFD model as a lean NASH model from the manuscript and revised the Discussion section as follows:**

**(Page 18, line 263-268) The difference between clinical studies and our study might be because of the difference in targets; clinical studies were performed on obese patients with NASH/NAFLD, setting a lower limit on body mass index or waist circumference [16, 17, 26]. Our study using the NASH model without obesity may indicate that TRF is not therapeutic for patients with NASH/NAFLD without obesity.**

**We also revised the Materials and Method section and figure captions to clarify that food consumption was measured per cage (three mice per cage), not per mouse.**

**(Page 7, line 85-87) mice were housed in groups of three per cage in a 12 h-12 h light-dark cycle (LD 12:12; lights on at Zeitgeber time (ZT) 0 and lights off at ZT12) for 2 weeks.**

**(Page 12, line 162) (A) Daily calorie intake was measured and calculated for every cage.**

**The language of the manuscript has been checked by an English proofreading company, Editage, which has been declared in the Acknowledgments section.**

Journal Requirements:

When submitting your revision, we need you to address these additional requirements.

**1. Please ensure that your manuscript meets PLOS ONE's style requirements, including those for file naming. The PLOS ONE style templates can be found at 
https://journals.plos.org/plosone/s/file?id=wjVg/PLOSOne_formatting_sample_main_body.pdf and** 
[**https://journals.plos.org/plosone/s/file?id=ba62/PLOSOne_formatting_sample_title_authors_affiliations.pdf**](https://journals.plos.org/plosone/s/file?id=ba62/PLOSOne_formatting_sample_title_authors_affiliations.pdf)

**Response**

**We ensured that our manuscript meets PLOS ONE’s style requirement.**

**2. To comply with PLOS ONE submissions requirements, in your Methods section, please provide additional information regarding the experiments involving animals and ensure you have included details on (1) methods of sacrifice, (2) methods of anesthesia and/or analgesia, and (3) efforts to alleviate suffering.**

**Response**

**We added statements to the manuscript regarding methods of anesthesia and sacrifice.**

**(Page 7, line 92-95) After 8 weeks, the mice were weighed, and blood was obtained under inhalation anesthesia with isoflurane. After cervical dislocation, liver sections and epididymal white adipose tissue (eWAT) were weighed and rapidly frozen in liquid nitrogen or fixed in 10% neutrally buffered formalin.**

**3. Thank you for stating the following financial disclosure: "Japan Society for the Promotion of Science KAKENHI, Grant Number JP21K21273 and JP22K17845 (both to Tomoyuki Sato)".
 
Please state what role the funders took in the study.  If the funders had no role, please state: "The funders had no role in study design, data collection and analysis, decision to publish, or preparation of the manuscript."**

**If this statement is not correct you must amend it as needed.**

**Please include this amended Role of Funder statement in your cover letter; we will change the online submission form on your behalf.**

**The funders had no role in this study. We declared this in the cover letter.**

**4. PLOS ONE now requires that authors provide the original uncropped and unadjusted images underlying all blot or gel results reported in a submission’s figures or Supporting Information files. This policy and the journal’s other requirements for blot/gel reporting and figure preparation are described in detail at https://journals.plos.org/plosone/s/figures#loc-blot-and-gel-reporting-requirements and https://journals.plos.org/plosone/s/figures#loc-preparing-figures-from-image-files. When you submit your revised manuscript, please ensure that your figures adhere fully to these guidelines and provide the original underlying images for all blot or gel data reported in your submission. See the following link for instructions on providing the original image data: https://journals.plos.org/plosone/s/figures#loc-original-images-for-blots-and-gels. 
  
In your cover letter, please note whether your blot/gel image data are in Supporting Information or posted at a public data repository, provide the repository URL if relevant, and provide specific details as to which raw blot/gel images, if any, are not available. Email us at plosone@plos.org if you have any questions.**

**Original blot data are attached as “S2 fig” in the Supporting information. In the cover letter, we declared that original blot image data are provided in Supporting information section.**

**5. Please include captions for your Supporting Information files at the end of your manuscript, and update any in-text citations to match accordingly. Please see our Supporting Information guidelines for more information:** [**http://journals.plos.org/plosone/s/supporting-information**](http://journals.plos.org/plosone/s/supporting-information)**.**

**We have revised our manuscript to include captions for the Supporting Information at the end of the manuscript (Page 22, line 377).**

**6. Please review your reference list to ensure that it is complete and correct. If you have cited papers that have been retracted, please include the rationale for doing so in the manuscript text, or remove these references and replace them with relevant current references. Any changes to the reference list should be mentioned in the rebuttal letter that accompanies your revised manuscript. If you need to cite a retracted article, indicate the article’s retracted status in the References list and also include a citation and full reference for the retraction notice.**

**We have reviewed and confirmed that the reference list is complete, correct, and does not include any retracted papers.**

**5. Review Comments to the Author**

Please use the space provided to explain your answers to the questions above. You may also include additional comments for the author, including concerns about dual publication, research ethics, or publication ethics. (Please upload your review as an attachment if it exceeds 20,000 characters)

**Reviewer #1: The manuscript by Sato et al focuses on the effect of time-restricted feeding on the murine NASH model. The topic is state of the art and the manuscript is clearly written for a non-expert. Figures support the text and the results are discussed in relation to the literature. I have no criticism, except that it would be interesting to know the reason for the 12 h restriction (why not e.g. 2*4 h).**

**We aimed to test the effect of time restriction of food intake in the active phase. In this study, we performed 8-h time-restricted feeding according to our previous paper (1). We used this method because recent reports indicate that continuous fasting time is important for anti-obesity effects.**

1.	Yasumoto Y, Hashimoto C, Nakao R, Yamazaki H, Hiroyama H, Nemoto T, et al. Short-term feeding at the wrong time is sufficient to desynchronize peripheral clocks and induce obesity with hyperphagia, physical inactivity and metabolic disorders in mice. Metabolism. 2016;65(5):714-27.

**Reviewer #2: The authors have performed a study of the effects of 8 weeks of active phase time-restricted feeding (TRF) in a non-obese model of rapidly-induced, advanced NAFLD (CDAHFD). End point analyses included measurements of liver and epidydimal adipose tissue weights, plasma ALT, AST and lipids, liver lipids, liver expression of inflammation and fibrosis genes, and histology for fibrosis. The authors conclude that TRF has limited suppressive effects in non-obese NASH. Given the limited applicability of the model used to human disease, this conclusion is too strong.
1) Statement at the end of the abstract: “Our results indicated that TRF has a limited suppressive effect in non-obese patients with NASH.” This needs to be revised to reflect that the work was done in a mouse model.**

**We revised the abstract to avoid making reference to human NASH as follows:**

**(Page 3, line 27-29) Here, we determined whether TRF affects NASH pathology induced by a choline-deficient high-fat diet (CDAHFD), which does not involve obesity**

**(Page 3, line 37-38) Our results indicated that TRF has a limited suppressive effect on CDAHFD-induced NASH pathology.**

**2) Was food consumption tracked during the active phase, i.e. was all food that was provided consumed within the ZT14-22 window?**

**Feeding was time-imposed in our experiment; food was accessible *ad libitum* to mice during ZT14 to ZT22 and was withdrawn from the cages at ZT22. To clarify this point, we revised the Materials and Methods section as follows:**

**（Page 7, line 88-91） others were fed CDAHFD (A06071302, 60 kcal% fat with 0.1% methionine and no added choline, Research Diets, Inc., New Brunswick, NJ, USA) ad libitum (AL) or under time-imposed restriction where food was accessible to mice during the active phase between ZT14-22 (TRF) for 8 weeks.**

**3) Fig. 1: Please provide caloric intake per day, rather than g per day, as it allows for easier determination of whether feeding protocols were isocaloric. Based on the data provided, it appears that there was likely some caloric restriction with this TRF protocol, so this study may be assessing TRF in combination with modest caloric restriction. Would the authors be able to provide AUCs for caloric intake?**

**Calorie intake was calculated and is shown in Fig 1A. Statistical tests revealed that there was a significant difference between AL and TRF in total calorie intake. Therefore, we revised the Results section and Discussion section as follows:**

**(Page 12, lie 153-154) TRF decreased calorie intake by 8% and ameliorated the increase in body weight observed in the CDAHFD-AL group.**

**(Page 16, line 218-219)** **TRF decreased total calorie intake by 8% and suppressed the increase in body weight (Fig 1A, 1B).**

**4) The authors conclude in the discussion (lines 213-215) that TRF did not decrease total food intake, but suppressed the increase in body weight, therefore indicating that energy expenditure was enhanced by TRF. There is not sufficient evidence provided to support this statement.**

**Energy expenditure was not measured. We toned down the statements in our conclusions to make it clear that this was merely a hypothesis suggested by some reports but not confirmed by experiments.**

**(Page 16, line 219-224) Considering that fasting enhances lipolysis in adipose tissue through activation of adipose triglyceride lipase and hormone-sensitive lipase [20], the lower eWAT weight in the TRF group may be due to sufficient length of the fasting period (16 h a day), in addition to calorie restriction. Moreover, the suppression of body weight gain is consistent with some reports that TRF enhances thermogenesis in adipocytes and energy expenditure [14, 21].**

**5) Please clarify the statement (lines 215-217): “Considering that a HFD narrows the fasting period, suppression of the increase in eWAT weight by TRF could be caused by fasting-triggered lipolysis.”**

**We revised our manuscript as follows:**

**(Page 16, line 219-224)** **Considering that fasting enhances lipolysis in adipose tissue through activation of adipose triglyceride lipase and hormone-sensitive lipase [20], the lower eWAT weight in the TRF group may be due to sufficient length of the fasting period (16 h a day), in addition to calorie restriction. Moreover, the suppression of body weight gain is consistent with some reports that TRF enhances thermogenesis in adipocytes and energy expenditure [14, 21].**

**6) In lines 254-256 the authors state: “Our results indicate that TRF has a suppressive effect on overnutrition-triggered steatosis mediated by the suppression of hepatic de novo lipogenesis (as documented in [14]), but not on steatosis triggered by the dysfunction of lipid export from the liver, independent of hepatic de novo lipogenesis.” In what context does steatosis triggered by the dysfunction of liver lipid export reflect human NAFLD? A lean human would not likely have a defect in lipoprotein secretion, unless in the setting of a genetic mutation. It is difficult to reconcile the CDAHFD model with any form of human NAFLD.**

**Response**

**The statement was based on the fact that choline is necessary to maintain hepatic function and that insufficiency in choline intake such as total parenteral nutrition often leads to fatty liver** **(1).**

**However, the choline deficiency model does not necessarily mimic lean NASH pathology** **(2). Hence, as the reviewer pointed out, it was inappropriate to consider the CDAHFD model as a human NASH model. We revised our manuscript as follows, simply referring to the difference between NASH induced by CDAHFD and that suffered by patients with obesity.**

**(Page 18, line 263-268) The difference between clinical studies and our study might be because of the difference in targets; clinical studies were performed on obese patients with NASH/NAFLD, setting a lower limit on body mass index or waist circumference [16, 17, 26]. Our study using the NASH model without obesity may indicate that TRF is not therapeutic for patients with NASH/NAFLD without obesity.**

1.	Zeisel SH. Choline: an essential nutrient for humans. Nutrition. 2000;16(7-8):669-71.

2.	Xu R, Pan J, Zhou W, Ji G, Dang Y. Recent advances in lean NAFLD. Biomed Pharmacother. 2022;153:113331.
